# Knowledge, Attitude, Practices, and Preparedness of Dental Professionals in Prescribing Nicotine Replacement Therapy

**DOI:** 10.1155/2022/5782228

**Published:** 2022-02-17

**Authors:** Pratibha Taneja, Parul Kashyap, Charu Mohan Marya, Ruchi Nagpal, Sakshi Kataria, Soumya Mahapatra, Anand Marya

**Affiliations:** ^1^Department of Public Health Dentistry, Sudha Rustagi College of Dental Sciences and Research, Haryana, India; ^2^Department of Orthodontics, Faculty of Dentistry, University of Puthisastra, Phnom Penh, Cambodia; ^3^Center for Transdisciplinary Research, Saveetha Dental College, Saveetha Institute of Medical and Technical Science, Saveetha University, Chennai, India

## Abstract

**Objective:**

To assess the knowledge, practice, attitude, and preparedness of dental professionals in prescribing nicotine replacement therapy (NRT). *Methodology*. A prevalidated voluntary web-based questionnaire was generated as a link through Google Drive and was sent to 117 dental professionals in North India using Whatsapp, Messenger, and Instagram social media platforms. A total of 94 responses were received and out of which 76 responses were analyzed (18 forms were excluded due to incomplete or duplicate responses). Frequency analysis was done using SPSS software version 21.

**Result:**

The participation rate was found to be 80.3%. More than half of the study population were familiar with the term NRT (77.6%) and its uses (67.1%), but approximately less than half of the total study subjects knew the duration (32.9%), cost (27.6%), dosage (25%), and contraindications (36.8%) of the NRT. Approximately 56.6% of the study participants showed a positive attitude towards helping patients to quit smoking through tobacco cessation counseling. Nearly one-fourth of the study population, i.e., 27.6%, were confident in explaining the negative impacts of tobacco, while 22.4% knew about the tobacco cessation protocol. Among the participants, only 27.6% reported that they practice NRT and out of which approximately less than 20% of the study participants were prescribing correct dose of NRT.

**Conclusion:**

Though study subjects had an ample knowledge regarding NRT use in tobacco cessation, it does not reflect their current attitude and preparedness. Thus, there is a need for continuing education to further train dental professionals for prescribing NRT.

## 1. Introduction

Tobacco kills more than 8 million people each year. More than 7 million of these deaths are due to the direct use of tobacco, and approximately 1.2 million are due to passive smoking or third handsmoke [1]. It causes a huge amount of suffering for individuals and their families by diminishing their quality of life and imparting an economic burden on them. More countries are making tobacco control a priority for saving lives by using the WHO MPOWER strategy, i.e., monitoring tobacco use and prevention policies, protecting people from tobacco smoke, offering help to quit tobacco use, warning about the danger of tobacco, enforcing bans on tobacco advertising, promotion, and sponsorship, and raising tobacco taxes [2].

The government of India launched the National Tobacco Control Program (NTCP) in the year 2007-08 to create awareness about the harmful effects of tobacco consumption, to reduce the production and supply of tobacco products, and to ensure effective implementation of the provisions under “The Cigarettes and Other Tobacco Products (Prohibition of Advertisement and Regulation of Trade and Commerce, Production, Supply, and Distribution) Act, 2003” (COTPA) to help the people quit tobacco use. Currently, the program is being implemented in all 36 states/union territories covering around 612 districts across the country. Under this program, training on tobacco cessation was provided to all health care providers including dental professionals. So far in India, a clinical protocol for the treatment of nicotine dependence has not been established by the health authorities. Western countries like Australia and the USA have clinical guidelines and procedures for the management of nicotine-dependent patients amended by their govt. [1].

In general, there are two main interventions by which professionals can facilitate tobacco cessation, i.e., by pharmacological and nonpharmacological approaches, yet the efficacy of pharmacotherapy gets multiplied when given synergistically with behavioral treatments [3].

Tobacco use is often perceived as a personal choice, but in fact, when tobacco users get fully aware of its health impact, most tobacco users want to quit but find it difficult to stop due to the addictive properties associated with nicotine. According to Albert et al. [4], approximately 50% of smokers visit a dentist annually which allows the dental provider to associate cessation advice with readily visible changes in oral status. Dental professionals are a largely untapped resource for providing provisional information about tobacco use, advice, and brief counseling to quit, and there are good reasons to believe that they can be effective in this role [5].

Useful, relevant, and important research to support the possible establishment of curricular guidelines aimed at training future dental professionals in the treatment and cessation of smoking. Equally important is to assist in the establishment of proposals to offer training to dental professionals that already work in private clinics/offices.

Dentists are in a unique position to provide salient, proximal information about tobacco use and oral and general health that can motivate tobacco users to quit [6]. Worldwide data showed that in Hong Kong, only 38% of the dentist had assisted patients in tobacco cessation, and in the United Kingdom (UK), 68% of the UK dentists and 75% of Northern Irish dentists felt obligated to provide tobacco cessation services. In India, the evidence related to the preparedness of dentists in prescription of nicotine replacement therapy (NRT) in dental settings is lacking that is why the present study was initiated to assess the knowledge, practices, attitude, and preparedness of dental professionals in prescribing NRT [5].

## 2. Methodology

### 2.1. Survey Design

This study was a snowball sampling-based online survey, written in accordance with the Checklist for Reporting Results of Internet E-surveys (CHERRIES) guidelines [6]. Participation in the survey was voluntary, with neither reward nor penalty. All respondents were informed that they were free to continue or quit at any time, and the submission of the questionnaire would be regarded as a consent to participate. To protect respondent privacy, the survey was anonymous, and all the raw data were stored in the authors' computer and kept confidential [6]. Ethical approval was sought from Institution Ethical Committee on Dec 5, 2020. We assured participants' confidentiality and also duplicate entries were avoided by preventing users' same IP address access to the survey twice. The data was collected from 05^th^ of December till 17^th^ of December 2020.

### 2.2. Survey Instrument

A 42-item questionnaire was prepared through the Google survey tool Gmail which was circulated to dentists in the North India region through Whatsapp and Instagram. We chose to distribute the survey through convenience and snowball sampling routes. The questionnaire comprised five sections.  Section 1 included general questions like name, age, years of experience in dental practice, and type of practice.  Section 2 comprised of 16 questions assessing the knowledge regarding NRT.  Section 3 had 5 questions for attitude assessment regarding NRT use. Sections 4 and 5 contained 13 questions in total to assess practices and preparedness of NRT use.

### 2.3. Development, Pretesting, and Recruitment

The reliability of the survey was assessed and was found to be acceptable (Cronbach′s *α* = 0.87). Firstly, the survey instrument was sent to experts in the field to give their opinion with respect to its simplicity, relativity, and importance; after that, it was sent to dental professionals.

Each survey item was reviewed by two groups of people. From the expert on questionnaire construction, common errors like confusing, leading, or double-barreled questions were confirmed and were modified.

Due to the specific prevention recommended during the outbreak, including prevention in close contacts and touch precautions, we designed an online questionnaire survey to follow these recommendations. Survey was sent as a web link which was created using Google Drive along with a text stating the title and the proposal of the study. We first disseminated the questionnaire link through Whatsapp, Gmail, and Instagram which have been the most widely used social media platforms in India.

The participants were encouraged to roll out the survey to as many people as possible. Thus, the link was forwarded to dental professionals apart from the first point of contact and so on. On receiving and clicking the link, the participants got autodirected to the information about the study.

### 2.4. Survey Administration

The questionnaire was collected using a professional online survey platform (Google survey tool of Gmail).

### 2.5. Response Rates

The questionnaire was sent to approx. 117 individuals, and among those, a total of 94 responses were received and out of which 76 responses were analyzed (18 forms were excluded due to incomplete or duplicate responses). As planned prior to commencing the survey, questionnaires with incomplete responses, apparent errors, and atypical timestamps were considered invalid and excluded. The results of the survey are reported according to the CHERRIES guidelines for reporting results of Internet E-survey.

### 2.6. Statistical Analysis

Descriptive statistics were performed to summarize the characteristics and the answers of each question. Categorical data were presented as counts and percentages. All the statistical analyses were performed by using the Statistical Package for the Social Sciences (SPSS Inc., Chicago, Illinois, USA) version 21. The statistical significance level was set at *p* < 0.05.

## 3. Results

### 3.1. Participant Characteristics and Educational Experiences

A total of 94 responses were received and out of which 76 responses were analyzed, yielding a response rate of 80.3%. Results of the present study showed that majority of the study participants belonged to 21-30 years old age group having <2 years (61.8%) of professional experience and were into clinical practice as well as were academicians.

### 3.2. Knowledge Assessment

Results showed that a total of 77.6% of the participants were familiar with the term NRT but only 25.0% reported to have the knowledge about the dosage and only 27.6% knows about the cost of NRT. A total of 46% of the study participants knew that NRT can be delivered in the gum forms. More than 70% of the respondents were unaware about nicotine dosage and duration of action (Table 1).

### 3.3. Attitude Assessment

Among the total, only 0.4% participants were interested in learning about smoking cessation counseling agreed that NRT is helpful in quitting tobacco. Majority of dentists also believed that tobacco cessation counseling by dentist could assist patients to quit smoking (56.6%), 42.1% did not consider tobacco cessation counseling as a part of dentist professional role, whereas 21.1% believed NRT increase the rate of successfully quitting the habit and only 34.2% believed that tapering the nicotine dependence is better than immediate complete abstinence from nicotine (Table 2).

### 3.4. Preparedness Assessment

Among total participants, 27.6% were confident in explaining the negative impacts of tobacco usage, 22.4% know what the tobacco cessation protocol, and 19.7% prepared to help patients in tobacco cessation. Only 6.6% were confident in assisting tobacco users to quit with written information, and only 5.3% participants were prepared enough to introduce NRT to their patients (Table 3).

### 3.5. Practice of NRT

Results showed that very few of the study participants were actually practicing NRT on their patient. Though majority of the participants recorded the type of tobacco use in the patient's card/folder (81.6%), but only 23.7% practiced NRT on their patient. Approximately 19.7% prescribed 21 mg patch with either 2 mg gum or 1 mg lozenge if patients smoke more than 10 cigarettes after 1 hour of waking up; 10.5% prescribed bupropion hydrochloride 150 mg 60-tab pack. For the patients who have expressed a desire to quit smoking among those for only 14.5% participants, professionals prescribed varenicline (as tartrate) 500 micrograms 56-tab pack (Table 4).

## 4. Discussion

It is a well-known fact that pharmacotherapy given along behavioral therapy enhances the rate of quitting smoking. Dental professionals can play important role in preventing adverse oral health effects by promoting smoking cessation. According to Dolan et al., worldwide, up to half of all dental surgeons advise patients to quit tobacco and also explain about the methods of quitting [7]. Contrary to this, in India, most dentists or health care professionals in the field do not ask or suggest methods to quit tobacco. This can be attributed to the fact that in India, no separate education or training is given to dental professionals during their course for helping individuals to quit tobacco. No such exposure during this learning phase could lead to the passive tobacco cessation guidance or services offered by these professionals [8]. Hence, the present study was initiated with the aim to assess the actual knowledge, practice, attitude, and preparedness of dental professionals in prescribing nicotine replacement therapy (NRT) in India.

Results of the present study showed that more than half of the dental professionals participated in the study were familiar with the replacement therapy used for nicotine addiction, i.e., NRT, but only 1/3 of the study population had any idea about the duration of use, cost, dosage, and contraindications of the NRT indicating the fact that dental professionals were not having any apt idea of the usage of NRT for cessation purposes. Similar results were reported by Hu et al., in their study of knowing how to help tobacco users [9].

The results of our study also depicted that there was an urgent need for TCC-related education for dental students. According to Liu et al., considerable proportion of the students were not confident nor knowledgeable to prescribe NRT and carry out the effective long-term TCC [5]. In addition, more than half of the respondents underrated the importance of dental professionals in helping tobacco-using patients. Dental students might not understand what an important role they could take in helping patients to quit tobacco use.

Approximately 56.6% of the present study participants showed positive attitude towards helping patients to quit smoking through tobacco cessation counseling. Nearly, one-fourth of the study population (i.e., 27.6%) were confident in explaining the negative impacts of tobacco while 22.4% knew about the tobacco cessation protocol. Among the participants, only 27.6% reported that they practice NRT and out of which approximately less than 20% of the study participants were prescribing correct dose of NRT.

According to Severson et al. [10], Hastreiter et al. [11], and Tomar et al. [12] in their studies regarding dental office practice for tobacco users, only a minority of the respondents in their study maintained records or advocated tobacco cessation practices among their clients. According to Severson et al., 40% of all the smokers make some attempt to quit in response to some advice from the general population [10]. There were also several lacunae in the knowledge, attitudes, and practices of the surgeons that were discovered in the present research.

The results of our study showed that most of the dental professionals lack knowledge of NRT and also believed NRT increase the rate of successfully quitting the habit. Failures to quit tobacco deny physicians the opportunity of recommending the appropriate method of intervention, anticipating challenges during the stages of quitting, and enlisting the necessary clinical and social support. The majority of dental professionals evaluated in the present study lacked good knowledge and practices related to smoking cessation. This study has highlighted the areas of deficient knowledge, practices, attitude, and preparedness of dental professionals in India regarding the use of NRT for smoking cessation. Similar results were obtained by Uti and Sofola in a study among Nigerian dentists and dental students [13]. We have identified the barriers to good smoking cessation practice. These findings could further help in the evaluation and formulation of effective guidelines on smoking cessation and smoking education programs [10].

Despite thorough literature search, we could not find any similar study, so the results of this study cannot be compared with the other study. The closest comparison point we could find is the WHO-sponsored GHPS survey on dental students [14]. Our results were almost similar to the result of GHPS survey; i.e., most of the dental professionals were lacking the knowledge regarding tobacco cessation services.

According to Murthy and Subodh, only one-third of the study population were aware of behavioral therapy for tobacco cessation and only half of the study population was aware of NRT which was very much similar to the results obtained in the present study [15]. Like any other survey, our research had some strength and limitations.

Survey was circulated through “E” platform providing best service of anonymity. Sample was procured through snowball sampling leading to elevated generalizability of the research findings. Though the survey topic was novel and the target population chosen was wide, but lesser response rate and small sample size obtained opened a need to conduct further research on this.

## 5. Conclusion

In conclusion, there is an urgent need to train dental health professionals regarding tobacco cessation service during their graduation curriculum and continuing dental education on tobacco cessation services. Useful, relevant, and important research are needed to support the possible establishment of curricular guidelines aimed at training future dental professionals in the treatment and cessation of smoking. Equally important is to assist in the establishment of proposals to offer training to dental professionals that already work in private clinics/offices.

## Figures and Tables

**Table 1:** Knowledge assessment of study participants regarding NRT.

	Yes	No	Not completely
Are you familiar with the term NRT?	59 (77.6%)	8 (10.5%)	9 (11.8%)
Are you aware about uses of NRT?	51 (67.1%)	6 (7.9%)	19 (25.0%)
According to your wish, which of the following is the delivery form of NRT?			
(i) Gums	35 (46.0%)	5 (6.5%)	36 (47.3%)
(ii) Patches	19 (25%)	24 (31.5%)	33 (43.2%)
(iii) Lozenges	6 (7.8%)	45 (59.2%)	25 (32.8%)
(iv) Nasal spray	10 (13.1%)	46 (53.5%)	23 (42.1)
(v) Inhalers	12 (15.7%)	45 (59.2%)	19 (25%)
(vi) Intravenous	13 (17.1%)	50 (65.6%)	13 (17.1%)
(vii) Intramuscular	10 (13.1%)	42 (55.2%)	24 (31.3%)
Are you aware about dosage of NRT?	19 (25%)	32 (42%)	25 (32.9)
Are you aware about duration of NRT?	25 (32.9%)	29 (38.2%)	22 (28.9%)
Are you aware about cost of NRT?	21 (27.6%)	41 (53.9%)	14 (18.4%)
Are you aware about indications of NRT?	50 (65.8%)	10 (13.2%)	16 (21.2%)
Are you aware about contraindication of NRT?	28 (36.8%)	20 (26.3%)	28 (36.8%)
Are you aware about of organizations helping nicotine-dependent patients to quit?	36 (47.4%)	20 (26.3%)	20 (26.3%)
Are you aware about how NRT works?	35 (46%)	18 (23.7%)	23 (30.3%)
Are you aware about different brands of NRT?	22 (28.9%)	32 (42.1%)2	22 (28.9%)
Are you aware about any side effect associated with the use of NRT?	33 (43.4%)	24 (31.6%)	19 (25.0%)
Availability of NRT near/at their institute.	21 (27.6%)	42 (55.3%)	13 (17.7%)
Do you have knowledge about separate cell at their institute for counseling nicotine-dependent patients?	48 (63.2%)	15 (19.7%)	13 (17.1%)
Are you aware about NRT's recommendation for occasional smokers?	39 (51.3%)	23 (30.3%)	14 (18.4%)
Do you have any knowledge about pregnant smokers given NRT's?	17 (22.4%)	42 (55.3%)	17 (22.4%)
Are you aware that adhesive transdermal patches can be applied to both the trunk and upper arm?	37 (48.7%)	24 (31.6%)	15 (19.7%)

**Table 2:** Attitude assessment of study participants regarding NRT.

	Strongly agree	Agree	Neutral	Disagree	Strongly disagree
I do not consider TC counseling as a part of dentist professional role.	32 (42.1%)	24 (31.6%)	7 (9.2%)	9 (11.8%)	4 (5.3%)
I believe TC counseling by dentist could assist patients to quit smoking.	43 (56.6%)	30 (39.5%)	2 (2.6%)	0	1 (1.3%)
I believe NRT increase the rate of successful quitting the habit.	16 (21.1%)	46 (60.5%)	13 (17.1%)	1 (1.3%)	0
Tapering the nicotine dependence is better than immediate complete abstinence from nicotine.	26 (34.2%)	36 (47.4%)	9 (11.8%)	3 (3.9%)	0
Be interested in learning about smoking cessation counseling.	28 (36.8%)	39 (51.3%)	7 (9.2%)	1 (1.3%)	1 (1.3%)
Nicotine dependence treatment be offered or provided to all your habitual smoker patients.	14 (18.4%)	42 (55.3%)	15 (19.7%)	5 (6.6%)	0

**Table 3:** Preparedness assessment of study participants regarding NRT.

	Strongly agree	Agree	Neutral	Disagree	Strongly disagree
I am confident in explaining the negative impacts of tobacco usage.	21 (27.6%)	45 (59.2%)	7 (9.2%)	2 (2.6%)	1 (1.3%)
I am not confident in assisting tobacco users to quit with written information.	5 (6.6%)	17 (22.4%)	22 (28.9%)	26 (34.2%)	6 (7.9%)
I am well prepared to help patients in tobacco cessation.	15 (19.7%)	31 (40.8%)	23 (30.3%)	6 (7.9%)	19 (1.3%)
I understand tobacco has a role in the etiology of oral cancer.	1 (1.3%)	2 (2.6%)	3 (3.9%)	17 (22.4%)	53 (69.7%)
I know what the tobacco cessation protocol is.	17 (22.4%)	32 (42.1%)	20 (26.3%)	7 (9.2%)	0
I am not knowledgeable enough to introduce NRT for my patients.	4 (5.3%)	19 (25.0%)	19 (25.0%)	28 (36.8%)	6 (7.9%)

**Table 4:** Practice assessment of study participants regarding NRT.

	Yes	No	Do not know
I ask patients about their tobacco usage states at the first appointment.	64 (84.2%)	8 (10.5%)	4 (5.3%)
I record the type of tobacco use in the patient's card/folder.	62 (81.6%)	12 (15.8%)	2 (2.6%)
Do you practice NRT to your patient?	21 (27.6%)	44 (57.9%)	11 (14.5%)
I prescribe 2 mg gum or 1 mg lozenge or 14 mg patch if a patient smokes less than 10 cigarettes after 1 hour of waking up.	18 (23.7%)	23 (30.3%)	35 (46.1%)
I prescribe 21 mg patch with either 2 mg gum or 1 mg lozenge if a patient smokes more than 10 cigarettes after 1 hour of waking up.	14 (18.4%)	22 (28.9%)	40 (52.6%)
I prescribe 21 mg patch with either 2 mg gum or 1 mg lozenge if a patient smokes less than 10 cigarettes within one hour of waking up.	11 (14.5%)	26 (34.2%)	39 (51.3%)
I prescribe 21 mg patch with either 4 mg gum or 2 mg lozenge if a patient smokes more than 10 cigarettes within one hour of waking up.	15 (19.7%)	23 (30.3%)	38 (50.0%)
I prescribe varenicline (as tartrate) 500 micrograms 56-tab pack, 1 mg 28-tab pack and 56-tab pack, and starter pack of 11 × 500-microgram tabs with 14 × 1 mg for the patients who have expressed a desire to quit smoking.	11 (14.5%)	21 (27.6%)	44 (57.9%)
I prescribe bupropion hydrochloride 150 mg 60-tab pack for the patients who have expressed a desire to quit smoking.	8 (10.5%)	20 (26.3%)	48 (63.2%)

## Data Availability

All research-related data can be made available on request.

## References

[1] World Health Organization (2015). *WHO global report on trends in prevalence of tobacco smoking 2015*.

[2] Ekpu V. U., Brown A. K. (2015). The economic impact of smoking and of reducing smoking prevalence: review of evidence. *Tobacco Use Insights*.

[3] Chen D., Wu L. T. (2015). Smoking cessation interventions for adults aged 50 or older: a systematic review and meta-analysis. *Drug and Alcohol Dependence*.

[4] Albert D. A., Severson H., Gordon J., Ward A., Andrews J., Sadowsky D. (2005). Tobacco attitudes, practices, and behaviors: a survey of dentists participating in managed care. *Nicotine & Tobacco Research*.

[5] Liu D. C. Y., Ho T. C. Y., Duangthip D., Gao S. S., Lo E. C. M., Chu C. H. (2019). Dental students’ awareness, preparedness and barriers towards managing tobacco-using patients—a cross-sectional study. *International Journal of Environmental Research and Public Health*.

[6] Shah A., Jacobs D. O., Martins H. (2006). DADOS-survey: an open-source application for CHERRIES-compliant web surveys. *BMC Medical Informatics and Decision Making*.

[7] Dolan T. A., McGorray S. P., Grinstead-Skigen C. L., Mecklenburg R. (1997). Tobacco control activities in U.S. dental practices. *The Journal of the American Dental Association*.

[8] Saddichha S., Rekha D. P., Patil B. K., Murthy P., Benegal V., Isaac M. K. (2010). Knowledge, attitude and practices of Indian dental surgeons towards tobacco control: advances towards prevention. *Asian Pacific Journal of Cancer Prevention*.

[9] Hu S., Pallonen U., McAlister A. L. (2006). Knowing how to help tobacco users: dentists’ familiarity and compliance with the clinical practice guideline. *The Journal of the American Dental Association*.

[10] Severson H. H., Eakin E. G., Stevens V. J., Lichtenstein E. (1990). Dental office practices for tobacco users: independent practice and HMO clinics. *American Journal of Public Health*.

[11] Hastreiter R. J., Bakdash B., Roesch M. H., Walseth J. (1994). Use of tobacco prevention and cessation strategies and techniques in the dental office. *Journal of the American Dental Association (1939)*.

[12] Tomar S. L., Husten C. G., Manley M. W. (1996). Do dentists and physicians advise tobacco users to quit?. *The Journal of the American Dental Association*.

[13] Uti O. G., Sofola O. O. (2011). Smoking cessation counseling in dentistry: attitudes of Nigerian dentists and dental students. *Journal of Dental Education*.

[14] Shah M. N. (2005). *Health Professionals in Tobacco Control: Evidence from Global Health Professional Survey (GHPS) of Dental Students in India*.

[15] Murthy P., Subodh B. N. (2010). Current developments in behavioral interventions for tobacco cessation. *Current Opinion in Psychiatry*.

